# Laboratory indices in patients with osteonecrosis of the femoral head: a retrospective comparative study

**DOI:** 10.1186/s13018-023-04235-0

**Published:** 2023-10-04

**Authors:** Haotian Zheng, Binglin Ye, Kesong Huang, Xiangming Gao, Wei Chen

**Affiliations:** 1 Second Affiliated Hospital of Heilongjiang University of Chinese Medicine, Harbin, China; 2 Department of Orthopaedics, Traditional Chinese Medical Hospital of Gansu Province, Qilihe District Guazhou Street 418, Lanzhou, 730050 Gansu China; 3 Gansu University of Chinese Medicine, Lanzhou, 730030 Gansu China; 4 Puyang Medical College, Henan Puyang, Henan, 457000 China

**Keywords:** Femoral head necrosis, Routine blood, Biochemical Indicators, Retrospective cases, Risk factors

## Abstract

**Background:**

Osteonecrosis of the femoral head is a degenerative condition linked to corticosteroids, alcoholism, or trauma. With its rising prevalence due to increased hormone drug use and its debilitating effects on young to middle-aged individuals, understanding its association with specific laboratory indicators can aid early diagnosis and prevention.

**Methods:**

Upon retrospective analysis of the clinical data pertaining to individuals diagnosed with femoral head necrosis, spanning from January 2016 to January 2022, a comprehensive evaluation was conducted within the same time frame. The study aimed to ascertain the presence of femoral head necrosis in a total of 1176 individuals. A total of 1036 healthy patients were recruited randomly, ensuring that their ages matched. The risk variables associated with the utilization of logistic regression analysis and analysis techniques are employed. The patient examines the age distribution within a specific age group.

**Results:**

The levels of high-density lipoprotein, low-density lipoprotein A1, lipoprotein B1, total protein, albumin, globulin, and other lipophilic metabolism and coagulation markers exhibited a statistically significant increase compared to the control group. A multifactor logistic regression analysis was conducted to identify potential risk factors associated with femoral head necrosis in patients.

**Conclusion:**

Femoral head necrosis is associated with a range of variables including coagulation malfunction, lipid metabolic abnormalities, and inflammation.

## Introduction

Osteonecrosis of the femoral head (ONFH) is a degenerative joint disorder that poses a considerable clinical obstacle within the field of orthopedics. The majority of individuals exhibit a co-occurrence of corticosteroid use, long-term drinking, or a history of trauma [1]. The prevalence rate in Japan is approximately 0.0182%, while in South Korea it is approximately 0.0289% [2]. The prevalence of new coronary pneumonia in recent years has raised concerns among researchers regarding the potential exacerbation of disease proportions resulting from the clinical administration of hormone drugs. This phenomenon has significant implications for quality of life, economic burden, and societal hygiene. The primary cause of femoral head necrosis is typically attributed to factors such as venous stasis inside the femoral head, compromised arterial blood flow, or disruption of bone cells and bone marrow constituents. The pain experienced in the hips and groin region is predominantly characterized by its intensity, causing discomfort to a significant extent. This condition is commonly observed among individuals within the age range of 20 to 59 years [3–5]. Research findings indicate that ONFH mostly arises from dysregulated lipid metabolism, impaired blood vessel function, aberrant adipocyte differentiation, and osteoporosis [6, 7]. Hence, a more comprehensive diagnosis of ONFH can be achieved through an examination of the interplay between laboratory markers, including inflammation, liver metabolism, lipid metabolism, and coagulation indicators.

This study examined the laboratory indicators of patients diagnosed with femoral head necrosis in a specific hospital. The study included a total of 1176 cases of femoral head necrosis and 1036 cases of non-femoral head necrosis, who sought medical examination during the same period. The study compared the laboratory indicators between these two groups, taking into account variations in age. This study aims to investigate the correlation between ONFH and various inflammatory indicators, liver metabolism indicators, lipid metabolism indicators, as well as coagulation indicators including liposuction. The objective is to identify laboratory indicators that can serve as representative markers for femoral head necrosis, thereby facilitating early clinical diagnosis and prevention strategies.

## Methods

### Participants

This study collected 1176 patients with femoral head necrosis in the Gansu Provincial Hospital of Traditional Chinese Medicine from January 2016 to January 2022. The clinical signs exhibited by patients are determined according to the "Chinese Adult Femoral Head Necrosis Clinical Diagnosis and Treatment Guidelines" published in 2020, as documented in the Chinese Orthopedic Magazine [8]. The primary symptom experienced by individuals is pain localized in the hip, hip joint, or groin area. In certain cases, this pain may be accompanied by knee discomfort. Furthermore, individuals may also observe limited hip internal rotation during activities involving the hip joint. Frequently encountered factors in career history include hip trauma, administration of corticosteroid drugs, a documented history of alcoholism, and considerations related to diversity. The study conducted at the Gansu Provincial Hospital of Traditional Chinese Medicine received approval from the Ethics Approval Committee (2021–0517).

Furthermore, the presence of the condition was verified using X-ray and magnetic resonance imaging (MRI) techniques. All individuals are encompassed by the diagnostic criteria for femoral head necrosis. From January 2015 to January 2022, a total of 1036 patients who sought medical evaluations at our institution were included in the control group for non-femoral head necrosis. No significant differences were seen among these patients. The inclusion criteria for this study are as follows: (1) The participants must meet the diagnostic criteria for necrosis of the femoral head as outlined in the Clinical Diagnosis and Treatment Code of the Femoral Head Necrosis. (2) Participants should not have received any specialist treatment prior to the collection of laboratory indicators. (3) A comprehensive set of laboratory tests including complete blood routine, biochemical, and coagulation series should be conducted prior to treatment. (4) Blood samples should be collected on the same day as the other assessments. The occurrence of blood seepage in individuals experiencing hunger, in the absence of clinical signs of infection such as fever, is observed. In this study, the researchers acquired informed consent from the patients and their families. The exclusion criteria for this study are as follows: (1) patients with concomitant malignant tumor diseases; (2) patients with severe diseases affecting vital organs; (3) patients with inadequate diagnosis and treatment records; (4) patients with mental disorders or altered consciousness; (5) patients with incomplete clinical data.

### Clinical data

The clinical data and laboratory indicators for this investigation were obtained from the medical records of both outpatient and inpatient individuals. Clinical data encompass fundamental information, such as gender, age, body weight index, and other relevant variables. Laboratory inspections encompass a range of blood routine, biochemical, and coagulation indicators. It is important to note that these aforementioned laboratory inspections are conducted under fasting conditions. Furthermore, the collection and treatment protocols for blood samples remain consistent across all procedures.

### Patients and public participation

The present investigation is characterized as a retrospective study. The medical records of patients have been collected over a span of five years. Consequently, it is not possible to get previous knowledge regarding patient priorities, experience, and preferences. This study involved the collection, analysis, and review of clinical data pertaining to patients. The involvement of patients in the recruitment and implementation of research has been lacking. In the initial stages, we disseminated our findings and reflections to those receiving medical treatment.

### Statistics analysis

The data analysis in this study was conducted using R version 4.1.3. Multiple interpolation approaches can be employed to address the issue of missing values in data insertion. To obtain baseline information and conduct differential analysis, non-positive continuous variables were described using the median and quartile values. The comparison between groups was performed using the Mann–Whitney U test. For categorical variables, the comparison between groups was conducted using the χ2 test, and the results were reported as samples and percentages. The application of single factor logistic analysis is utilized to examine the influence of clinical characteristics in both the case group and control group. The variables with a significance level of *P* < 0.05 are then incorporated into the multiple factor logistic analysis, indicating statistically significant differences.

## Result

### The basic situation of the research object

The study encompassed a sample size of 2202 individuals, with 1036 participants assigned to the case group and 1176 participants assigned to the control group. There were significant differences observed between the two groups in terms of age, gender, and BMI (*P* < 0.05) (Table 1). In relation to inflammatory cell indicators, all indicators except for eosinophil ratio (SBCR) showed significant differences between the two groups (*P* < 0.05) (Table 1). Furthermore, there were differences in the anemia indicators specifically related to red blood cell-specific volume (HCT) (*P* < 0.05) (Table 1). Similarly, with regard to coagulation indicators, all indicators except for prothrombin time (PT) exhibited significant differences between the two groups (*P* < 0.05) (Table 1). Lastly, among the kidney metabolic indicators, only CREA, uric acid (UA), MG +  + , and CA +  + did not show significant differences between the two groups, while the remaining indicators did (Table 1). Significant differences (*P* < 0.05) were observed in the liver metabolic indicators, with the exception of Alanine aminotransferase (ALT), between the two groups as shown in Table 1. Similarly, significant differences (*P* < 0.05) were found in most high blood lipid-related indicators, except for total cholesterol (TCHO), very low-density lipoprotein cholesterol (VLDL), and triglyceride (TG), which did not show significant differences between the two groups. These findings are summarized in Table 1. Significant changes (*P* < 0.05) were observed in the myocardial indicator, with the exception of α-hydroxybutyrate dehydrogenase (*α*-HBDH). The remaining indicators showed significant differences between the two groups (*P* < 0.05) (Table 1).

**Table 1:** The basic situation of the research object

Variable	Control group (*N* = 1036)	Clinical group (*N* = 1176)	*P*
*Age*			< 0.001
< 45	497 (47.973%)	258 (21.939%)	
45–60	414 (39.961%)	545 (46.344%)	
≥ 60	125 (12.066%)	373 (31.718%)	
*Sex:*			0.002
Male	638 (61.583%)	647 (55.017%)	
Female	398 (38.417%)	529 (44.983%)	
*BMI*			< 0.001
< 24	550 (53.089%)	498 (42.347%)	
≥ 24	486 (46.911%)	678 (57.653%)	
*Inflammatory cell index*			
WBC	5.970 [4.940;7.175]	5.650 [4.640;6.940]	< 0.001
MBCR	57.550 [51.025;64.300]	59.600 [53.100;66.500]	< 0.001
LBCR	32.450 [25.300;38.675]	30.500 [24.100;36.785]	< 0.001
DBCR	6.900 [5.900;8.100]	6.600 [5.600;8.000]	0.001
SBCR	1.700 [0.900;2.600]	1.600 [0.900;2.800]	0.756
JBCR	0.500 [0.300;0.700]	0.400 [0.200;0.600]	< 0.001
*Coagulation indicator*			
PLT	206.000 [167.000;254.000]	191.500 [147.750;243.000]	< 0.001
HCY	16.000 [13.000;21.000]	15.000 [13.000;19.000]	0.001
FDP	0.800 [0.500;1.500]	1.700 [1.000;3.600]	< 0.001
DD	0.170 [0.110;0.300]	0.405 [0.240;1.030]	< 0.001
APTT	25.900 [23.900;28.500]	25.800 [23.100;29.600]	0.865
PT	2.400 [2.100;2.800]	2.800 [2.330;3.505]	< 0.001
*Liver metabolism*			
TBIL	14.755 [10.845;19.775]	12.845 [9.725;17.248]	< 0.001
DBIL	3.460 [2.530;4.830]	3.300 [2.380;4.590]	0.005
IBIL	11.065 [8.300;15.115]	9.600 [7.010;12.690]	< 0.001
ALT	20.000 [14.000;32.000]	20.000 [14.000;31.000]	0.842
AST	20.000 [17.000;26.000]	19.000 [15.000;24.000]	< 0.001
AST/ALT	1.000 [0.700;1.400]	0.900 [0.700;1.200]	0.004
γ-GT	16.000 [11.000;28.000]	20.000 [13.000;35.000]	< 0.001
ALP	73.000 [59.000;92.000]	80.000 [65.000;98.000]	< 0.001
AMY	61.000 [49.000;78.000]	57.000 [45.000;70.000]	< 0.001
*Hyperlipidemia indicator*			
CHO/HDL	3.070 [2.620;3.630]	3.370 [2.898;3.990]	< 0.001
ApoA1	1.100 [0.970;1.250]	1.120 [0.970;1.320]	0.002
ApoB	0.620 [0.490;0.770]	0.680 [0.560;0.830]	< 0.001
Atherosclerotic index	1.660 [1.320;2.050]	1.740 [1.400;2.180]	< 0.001
TCHO	4.090 [3.520;4.770]	4.100 [3.500;4.800]	0.63
HDL-C	1.310 [1.160;1.510]	1.200 [1.030;1.380]	< 0.001
LDL-C	2.190 [1.730;2.707]	2.110 [1.690;2.567]	0.006
VLDL	0.610 [0.410;0.910]	0.630 [0.440;0.920]	0.106
TG	1.340 [0.920;2.010]	1.390 [0.960;2.007]	0.168

### Single factor analysis

In the examination of individual characteristics (Table 2), it was observed that patients aged 45–60 (OR: 2.534, 95%CI: 2.082–3.090, *P* < 0.001) and patients aged ≥ 60 (OR: 5.735, 95%CI: 4.467–7.402, *P* < 0.001) had a significantly higher risk of onset compared to patients aged < 45 years old. In comparison with women, men have a significantly higher risk of onset (OR: 1.310, 95%CI: 1.106–1.554, *P* = 0.002). In comparison with individuals with a BMI less than 24, patients with a BMI greater than 24 had a statistically significant odds ratio of 1.540 (95% confidence interval: 1.302–1.823, *P* value < 0.001). The likelihood of occurrence is elevated. The results indicate that there is a significant association between the inflammatory cell indicators and the risk of onset. Specifically, the carbon dioxide combining power (MBCR) (OR: 1.017, 95%CI: 1.009–1.025, *P* < 0.001) is positively correlated with the higher risk of onset. On the other hand, the white blood cell (WBC) (OR: 0.944, 95%CI: 0.906–0.983, *P* = 0.006), lymphocyte ratio (LBCR) (OR: 0.982, 95%CI: 0.974–0.991, *P* < 0.001), basophil ratio (JBCR) (OR: 0.344, 95%CI: 0.255–0.463 *P* < 0.001), and monocyte ratio (DBCR) (OR: 0.949, 95%CI: 0.909–0.992, *P* = 0.020) are negatively associated with the risk of onset. The high level of a certain factor is associated with a decreased risk of onset. Specifically, in the context of coagulation indicators, the presence of fibrinogen degradation products (FDP) (OR: 1.024, 95%CI: 1.010–1.037, *P* < 0.001), D-dimer (DD) (OR: 1.140, 95%CI: 1.081–1.203, *P* < 0.001), PT (OR: 1.035, 95%CI: 1.019–1.051, *P* < 0.001), and FIB (OR: 2.285, 95%CI: 2.019–2.586, *P* < 0.001) have been found to be significantly associated with a higher risk. There is a positive correlation between high levels of Jun protein and an increased likelihood of disease onset. The platelet (PLT) variable had an odds ratio (OR) of 0.998, with a 95% confidence interval (CI) of 0.997–0.999, and a *P *value of 0.001. Similarly, the homocysteine (HCY) variable had an OR of 0.984, with a 95% CI of 0.976–0.991, and a *P *value less than 0.001. The lower risk of onset is associated with liver metabolism indicators, namely γ-glutamyl transpeptidase (γ-GT) (OR: 1.007, 95%CI: 1.004–1.010, *P* < 0.001), which shows a strong correlation with a higher incidence risk. The results of the study indicate that there is a significant association between indirect bilirubin (IBIL) and the outcome variable, with an OR of 0.933 (95% confidence interval [CI]: 0.918–0.949, *P* < 0.001). Similarly, the analysis reveals a significant relationship between aspartate/alanine (AST/ALT) and the outcome variable, with an odds ratio of 0.885 (95% CI: 0.791–0.991, *P* = 0.034). Additionally, the study finds a significant correlation between AMY and the outcome variable, with an odds ratio of 0.994 (95% CI: 0.991–0.997, *P* < 0.001). The risk of onset is significantly higher in individuals with elevated levels of high blood lipid indicators, specifically Cho/high-density lipoprotein (HDL) (OR: 1.626, 95%CI: 1.461–1.810, *P* < 0.001), Apolipoprotein1 (APOA1) (OR: 2.263, 95%CI: 1.643 -3.117, *P* < 0.001), and Apolipoprotein B (APOB) (OR: 3.900, 95%CI: 2.612–5.823, *P* < 0.001). The risk of onset is significantly lower for individuals with higher levels of high-density lipoprotein cholesterol (HDL-C) (OR: 0.243, 95%CI: 0.181181–0.325, *P* < 0.001) and low-density lipoprotein cholesterol (LDL-C) (OR: 0.852, 95%CI: 0.758–0.958, *P* = 0.007), as shown in Table 2.

**Table 2:** Single factor analysis

Variable	OR[95%CI]	*P*
*Age*		
< 45	Ref.	Ref.
45–60	2.534 [2.082–3.090]	< 0.001
≥ 60	5.735 [4.467–7.402]	< 0.001
*Sex:*		
Male	Ref.	Ref.
Female	1.310 [1.106–1.554]	0.002
*BMI:*		
< 24	Ref.	Ref.
≥ 24	1.540 [1.302–1.823]	< 0.001
*Inflammatory cell index*		
WBC	0.944 [0.906–0.983]	0.006
MBCR	1.017 [1.009–1.025]	< 0.001
LBCR	0.982 [0.974–0.991]	< 0.001
DBCR	0.949 [0.909–0.992]	0.02
SBCR	1.033 [0.984–1.084]	0.185
JBCR	0.344 [0.255–0.463]	< 0.001
*Coagulation indicator*		
PLT	0.998 [0.997–0.999]	0.001
HCY	0.984 [0.976–0.991]	< 0.001
FDP	1.024 [1.010–1.037]	< 0.001
DD	1.140 [1.081–1.203]	< 0.001
APTT	1.035 [1.019–1.051]	< 0.001
PT	2.285 [2.019–2.586]	< 0.001
*Liver metabolism*		
TBIL	0.999 [0.996–1.002]	0.52
DBIL	0.972 [0.941–1.004]	0.09
IBIL	0.933 [0.918–0.949]	< 0.001
ALT	1.001 [0.998–1.005]	0.509
AST	0.998 [0.993–1.004]	0.57
AST/ALT	0.885 [0.791–0.991]	0.034
γ-GT	1.007 [1.004–1.010]	< 0.001
ALP	0.998 [0.997–1.000]	0.049
AMY	0.994 [0.991–0.997]	< 0.001
*Hyperlipidemia indicator*		
CHO/HDL	1.626 [1.461–1.810]	< 0.001
ApoA1	2.263 [1.643–3.117]	< 0.001
ApoB	3.900 [2.612–5.823]	< 0.001
Atherosclerosis index	0.993 [0.980–1.007]	0.338
TCHO	0.999 [0.996–1.003]	0.693
HDL-C	0.243 [0.181–0.325]	< 0.001
LDL-C	0.852 [0.758–0.958]	0.007
VLDL	0.992 [0.867–1.135]	0.902
TG	1.018 [0.950–1.091]	0.613

### Multifactor analysis

Age, gender, and body mass index (BMI) adjustments have been applied to all indicator groups.

The OR and corresponding 95% CI for the variables OR, JBCR, and DBCR were found to be 0.906 (95%CI: 0.857–0.957, *P* = 0.001), 0.255 (95%CI: 0.176–0.365, *P* < 0.001), and 0.878 (95%CI: 0.814–0.945, *P* = 0.001), respectively. These results indicate a strong association between these variables and a decreased probability of onset, as shown in Table 3.

**Table 3:** All indicator groups have undergone age, gender, and BMI adjustment

Variable	OR	95%CI		*P*
		Lower	Upper	
*Age*				
< 45	Ref.			Ref.
45–60	2.379	1.94	2.923	< 0.001
≥ 60	5.265	4.05	6.88	< 0.001
*Gender*				
Male	Ref.			Ref.
Female	1.033	0.854	1.249	0.736
*BMI*				
< 24	Ref.			Ref.
≥ 24	1.453	1.213	1.74	< 0.001
WBC	0.926	0.881	0.971	0.002
MBCR	0.915	0.867	0.965	0.001
LBCR	0.906	0.857	0.957	0.001
DBCR	0.878	0.814	0.945	0.001
JBCR	0.255	0.176	0.365	< 0.001

*WBC* White blood cell, *MBCR* Carbon dioxide combining power, *LBCR* Lymphocyte ratio, *DBCR* Monocyte ratio, *SBCR* Eosinophil ratio, *JBCR* Basophil ratio

In the analysis of coagulation indicators, it was found that DD (OR: 1.111, 95%CI: 1.045–1.192, *P* = 0.001), PT (OR: 1.043, 95%CI: 1.024–1.064, *P* < 0.001), and FIB (OR: 0.975–0.992, 95%CI: *P* < 0.001) were significantly associated with a decreased probability of onset, as shown in Table 4.

**Table 4:** Multifactor analysis of the coagulation indicator

Variable	OR	95%CI		*P*
		Lower	Upper	
*Age*				
< 45	Ref.			Ref.
45–60	2.181	1.757	2.71	< 0.001
≥ 60	4.643	3.525	6.143	< 0.001
*Gender*				
Male	Ref.			Ref.
Female	1.066	0.877	1.297	0.52
*BMI*				
< 24	Ref.			Ref.
≥ 24	1.539	1.276	1.857	< 0.001
PLT	0.997	0.995	0.998	< 0.001
HCY	0.983	0.975	0.992	< 0.001
FDP	0.987	0.968	1.003	0.128
DD	1.111	1.045	1.192	0.001
PT	1.043	1.024	1.064	< 0.001

*PLT* Platelet, *HCY* Homocysteine, *FDP* Fibrinogen degradation products, *DD* D-dimer, *APTT* Activated partial thromboplastin time, *PT* Prothrombin time

In the context of liver metabolic indicators, it was found that γ-GT (OR: 1.008, 95%CI: 1.005–1.011, *P* < 0.001) exhibited a higher risk of onset, while IBIL (OR: 0.920 95%CI: 0.903- 0.937, *P* < 0.001), AST/ALT (OR: 0.870, 95%CI: 0.756–0.972, *P* = 0.029), and Amy (OR: 0.993, 95%CI: 0.990–0.996, *P* < 0.001) were associated with a reduced risk of onset (Table 5).

**Table 5:** Multifactor analysis of the liver metabolic indicators

Variable	OR	95%CI		*P*
		Lower	Upper	
*Age*				
< 45	Ref.			Ref.
45–60	2.67	2.17	3.29	< 0.001
≥ 60	6.253	4.797	8.199	< 0.001
*Gender*				
Male	Ref.			Ref.
Female	1.098	0.905	1.33	0.343
*BMI*				
< 24	Ref.			Ref.
≥ 24	1.39	1.156	1.67	< 0.001
IBIL	0.92	0.903	0.937	< 0.001
AST/ALT	0.87	0.756	0.972	0.029
γ-GT	1.008	1.005	1.011	< 0.001
AMY	0.993	0.99	0.996	< 0.001

*IBIL* Indirect bilirubin, *ALT* Alanine aminotransferase, *AST* Aspartate aminotransferase, *AST/ALT* Aspartate/alanine, *Γ-GT* γ-glutamyl transpeptidase, *AMY* Amylase

In the context of elevated blood lipid markers, it was shown that APOA1 (odds ratio [OR]: 17.274, 95% confidence interval [CI]: 9.506–32.237, *P* < 0.001) and APOB (OR: 5.311, 95% CI: 2.351–12.003, *P* < 0.001) exhibited greater levels when compared. There is a significant high risk of onset associated with HDL-C (odds ratio [OR]: 0.037, 95% confidence interval [CI]: 0.018–0.072, *P* < 0.001) and LDL-C (OR: 0.667, 95% CI: 0.534–0.831, *P* < 0.001). The observed phenomenon is associated with a decreased likelihood of occurrence, as seen in Table 6.

**Table 6:** Multifactor analysis of the high blood lipid indicators

Variable	OR	95%CI		*P*
		Lower	Upper	
*Age*				
< 45	Ref.			Ref.
45–60	2.231	1.797	2.775	< 0.001
≥ 60	5.216	3.954	6.917	< 0.001
*Gender*				
Male	Ref.			Ref.
Female	1.409	1.151	1.726	0.001
*BMI*				
< 24	Ref.			Ref.
≥ 24	1.235	1.021	1.494	0.03
CHO/HDL	0.925	0.735	1.172	0.51
ApoA1	17.274	9.506	32.237	< 0.001
ApoB	5.311	2.351	12.003	< 0.001
HDL-C	0.037	0.018	0.072	< 0.001

*CHO/HDL* Very low-density lipoprotein, *APOA1* Apolipoprotein1, *APOB* Apolipoprotein B, *HDL-C* High-density lipoprotein cholesterol

### ROC curve analysis

**Fig. 1:**
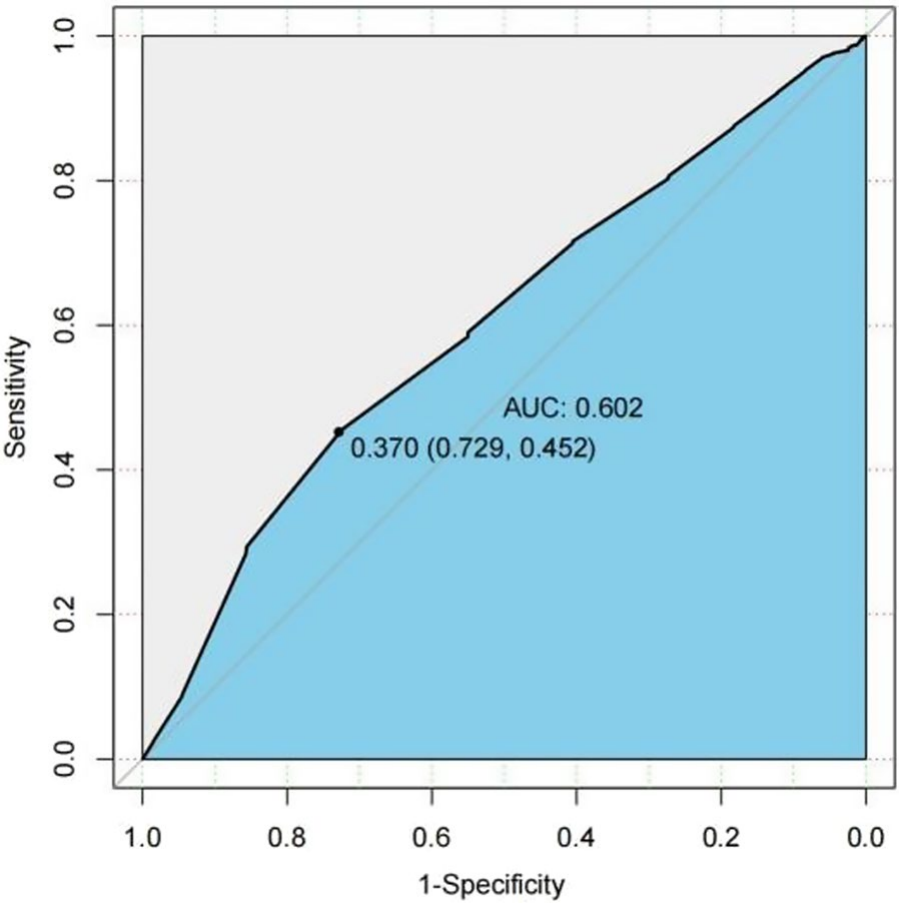
JBCR in the inflammatory cell indicators

**Fig. 2:**
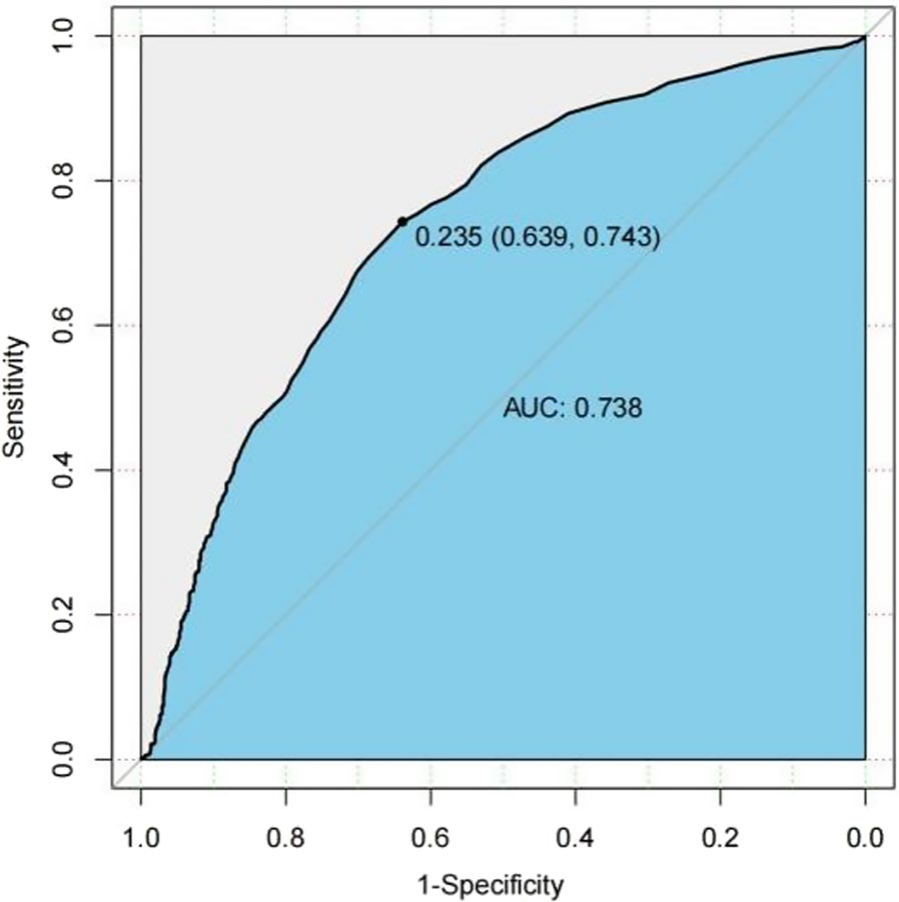
DD in the coagulation indicator

**Fig. 3:**
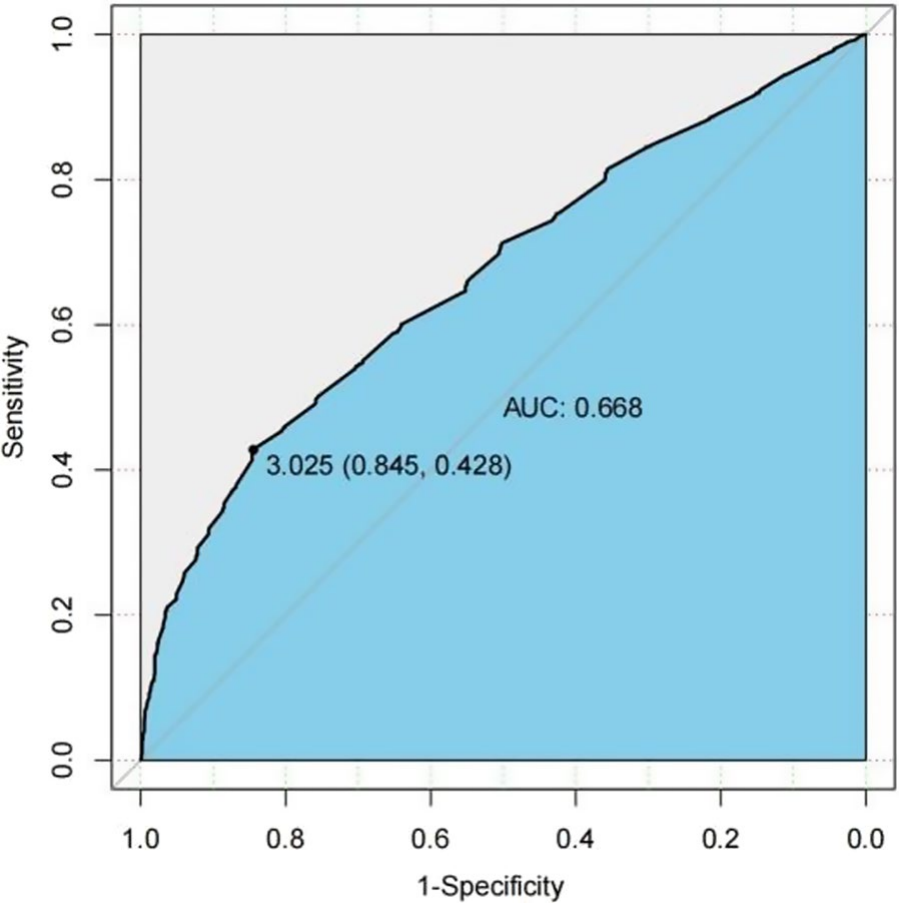
FIB in the coagulation indicator

**Fig. 4:**
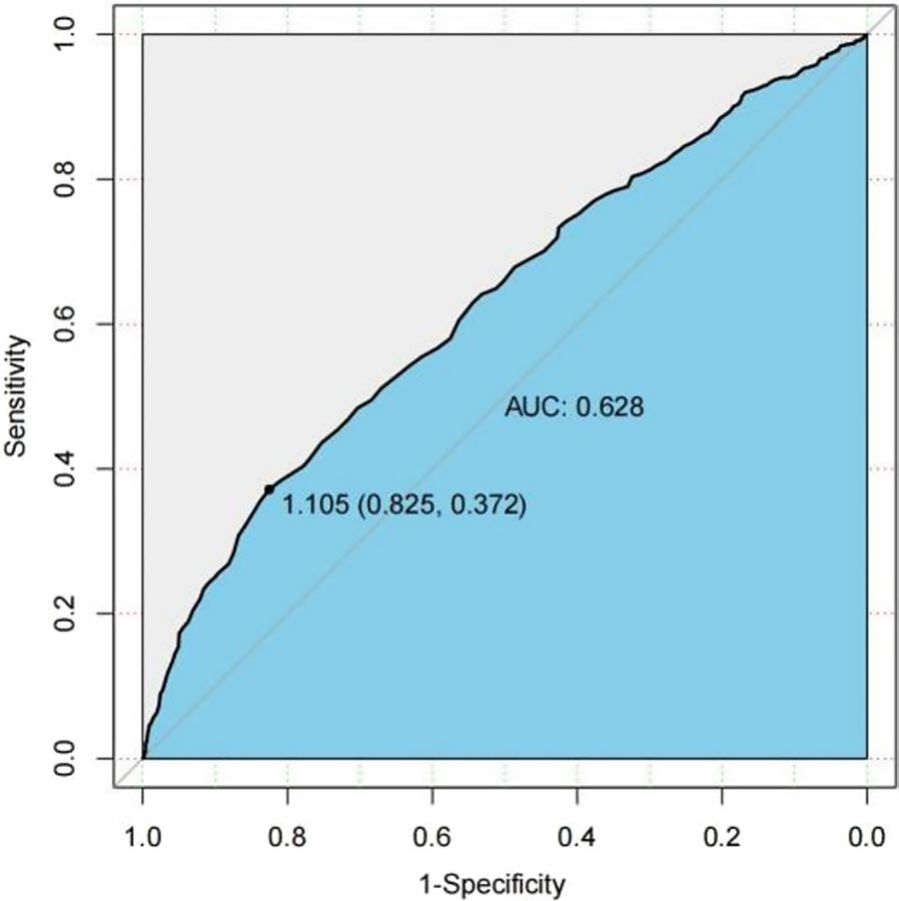
IBIL in the liver metabolic indicators

**Fig. 5:**
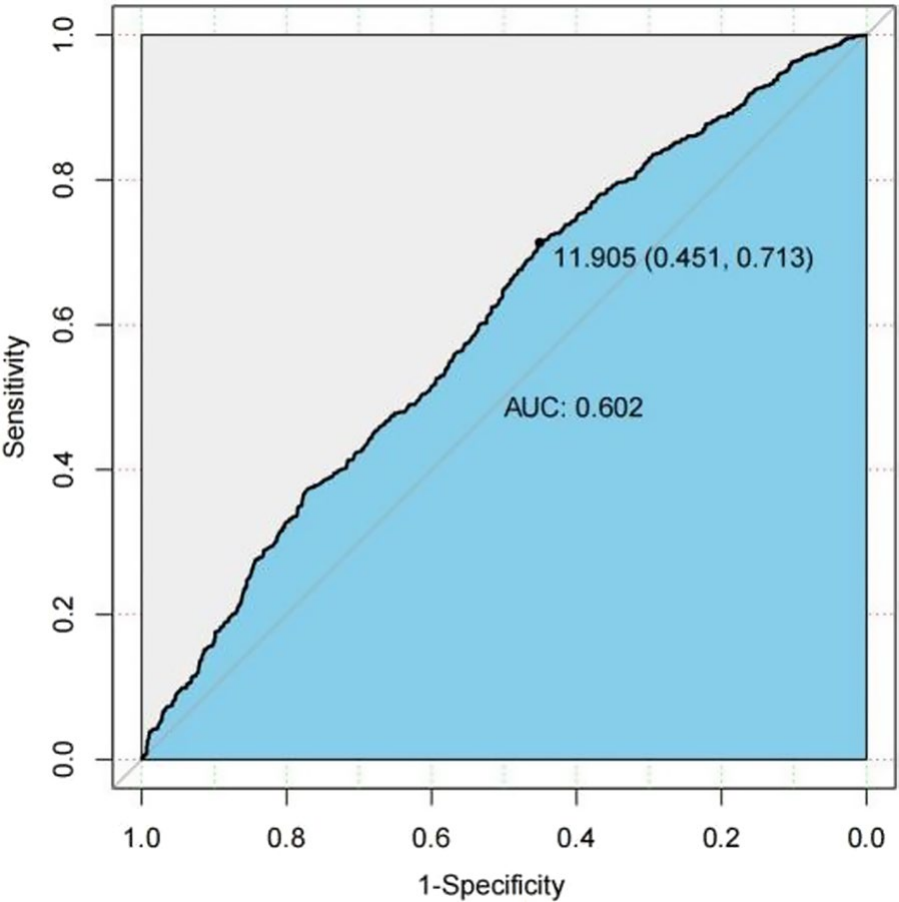
HDL-C in the blood lipid index

The analysis of the receiver operating characteristic (ROC) curve indicates that there is a significant correlation between certain variables and the onset of the condition. Specifically, age, JBCR (as shown in Fig. 1) in the inflammatory cell indicators, DD and FIB (as shown in Figs. 2 and 3) in the coagulation indicator, and IBIL in the liver metabolic indicators (as shown in Fig. 4) exhibit a high correlation with HDL-C in the blood lipid index (as shown in Fig. 5). The area under the curve (AUC) for these variables is greater than 0.6, indicating their predictive value in determining the onset of the condition. Among the many indices, the AUC value for DD reaches 0.738, indicating that DD potentially exhibits a favorable diagnostic performance for ONFH (Osteonecrosis of the Femoral Head).

### Subgroup analysis

In order to account for the variations in age, gender, and BMI between the two groups, this study employs a subgroup analysis to examine the aforementioned indicators. This analysis is conducted using the multifactor logistic analysis method previously indicated in the study. The subsequent investigation aims to examine the impact of certain indications of ONFH on the diagnostic process of ONFH. The findings indicate a negative correlation between greater JBCRs and the likelihood of onset in each subgroup across various subgroups. Conversely, higher FIB levels are positively associated with an increased risk of start. Additionally, higher PLT levels are positively correlated with a higher risk of beginning. There is a correlation between lower risk of onset and the presence of liver metabolic markers in each Asian ethnicity. There is a positive correlation between elevated levels of γ-GT and an increased likelihood of disease onset. There is a negative correlation between IBIL levels and the risk of onset. Within each Asian group, there is a positive correlation between APOA1 levels and the risk of onset, whereas there is a negative correlation between HDL-C levels and the risk of onset, as seen in Table 7.

**Table 7:** Subgroup analysis

Group	Variable	Age group			
		Age < 45			
		OR	95%CI		*P*
			Lower	Upper	
Liver	Sex	1.182	0.827	1.689	0.358
	BMI	1.022	0.987	1.057	0.219
	IBIL	0.919	0.888	0.949	0
	AST/ALT	0.83	0.633	1.006	0.116
	γ-GT	1.007	1.003	1.012	0.001
	AMY	0.982	0.974	0.99	0
Hyperlipidemia	Sex	1.987	1.361	2.911	0
	BMI	1.009	0.973	1.045	0.625
	CHO/HDL	0.59	0.355	0.982	0.042
	ApoA1	24.85	7.609	89.853	0
	ApoB	72.783	13.259	420.849	0
	HDL-C	0.007	0.002	0.031	0
	LDL-C	0.751	0.492	1.134	0.178
Coagulation	Sex	1.134	0.787	1.632	0.498
	BMI	1.043	1.008	1.082	0.018
	PLT	0.994	0.992	0.997	0
	HCY	0.986	0.97	1.001	0.085
	FDP	0.872	0.781	0.955	0.008
	DD	1.847	1.275	2.914	0.004
	PT	1.055	1.023	1.091	0.001
	FIB	2.536	1.991	3.277	0
Inflammatory cells	Sex	1.11	0.791	1.553	0.542
	BMI	1.036	1.003	1.071	0.035
	wbc	0.896	0.82	0.974	0.012
	mbcr	0.931	0.847	1.024	0.141
	lbcr	0.915	0.829	1.009	0.074
	dbcr	0.867	0.76	0.985	0.031
	jbcr	0.272	0.134	0.539	0

Group	Variable	Age group			
		45–60			
		OR	95%CI		*P*
			Lower	Upper	
Liver	Sex	1.173	0.891	1.548	0.257
	BMI	1.029	0.996	1.065	0.087
	IBIL	0.913	0.888	0.937	0
	AST/ALT	0.845	0.637	0.999	0.14
	γ-GT	1.006	1.002	1.011	0.011
	AMY	0.996	0.99	1	0.07
Hyperlipidemia	Sex	1.392	1.042	1.864	0.026
	BMI	1.014	0.98	1.05	0.442
	CHO/HDL	0.879	0.656	1.199	0.402
	ApoA1	14.334	6.287	34.452	0
	ApoB	4.541	1.538	13.385	0.006
	HDL-C	0.043	0.016	0.109	0
	LDL-C	0.644	0.471	0.877	0.005
Coagulation	Sex	1.098	0.831	1.451	0.512
	BMI	1.039	1.005	1.077	0.03
	PLT	0.998	0.996	1	0.066
	HCY	0.982	0.967	0.995	0.01
	FDP	0.985	0.959	1.006	0.184
	DD	1.093	1.017	1.191	0.02
	PT	1.033	1.005	1.063	0.022
	FIB	1.882	1.567	2.281	0
Inflammatory cells	Sex	0.992	0.755	1.303	0.952
	BMI	1.033	1	1.069	0.054
	wbc	0.941	0.875	1.006	0.09
	mbcr	0.903	0.838	0.971	0.008
	lbcr	0.908	0.84	0.978	0.015
	dbcr	0.883	0.795	0.98	0.021
	jbcr	0.226	0.133	0.376	0

Group	Variable	Age group			
		Age ≥ 60			
		OR	95%CI		*P*
			Lower	Upper	
Liver	Sex	0.816	0.526	1.259	0.361
	BMI	1.112	1.044	1.191	0.002
	IBIL	0.931	0.894	0.969	0
	AST/ALT	1.046	0.767	1.542	0.8
	γ-GT	1.019	1.006	1.035	0.011
	AMY	0.995	0.99	1	0.036
Hyperlipidemia	Sex	0.989	0.622	1.57	0.962
	BMI	1.107	1.036	1.19	0.004
	CHO/HDL	1.525	0.91	2.672	0.123
	ApoA1	19.583	5.768	74.239	0
	ApoB	0.3	0.048	1.884	0.197
	HDL-C	0.096	0.024	0.363	0.001
	LDL-C	0.715	0.427	1.178	0.193
Coagulation	Sex	0.735	0.464	1.156	0.186
	BMI	1.155	1.078	1.244	0
	PLT	0.997	0.994	0.999	0.013
	HCY	0.979	0.962	0.995	0.013
	FDP	1.079	1.012	1.183	0.052
	DD	1.019	0.809	1.196	0.825
	PT	1.031	0.985	1.085	0.212
	FIB	1.985	1.468	2.736	0
Inflammatory cells	Sex	0.955	0.615	1.481	0.839
	BMI	1.118	1.048	1.199	0.001
	wbc	0.947	0.842	1.07	0.374
	mbcr	0.91	0.783	1.044	0.196
	lbcr	0.883	0.757	1.018	0.1
	dbcr	0.844	0.695	1.013	0.076
	jbcr	0.298	0.135	0.642	0.002

## Discussion

The etiology of bone injury remains incompletely elucidated; nevertheless, existing research suggests that inflammatory markers, lipid metabolism markers, coagulation markers, and indicators of liver function metabolism are deemed significant in the context of ONFH. These indicators are believed to exert a pivotal influence at various phases of the disease. Numerous studies have demonstrated that trauma, hormonal imbalances, and alcohol consumption are associated with a decline in osteosomial levels, an elevation in high internal pressure, an increase in adipose tissue accumulation, and a reduction in blood flow rate, ultimately culminating in ONFH [6, 7, 9, 10].

There are multiple factors that might contribute to blood circulation abnormalities, including hyperlipidemia, lipid metabolic diseases, and impaired coagulation function, among others. Numerous investigations have demonstrated that hyperlipidemia constitutes a risk factor for ONFH. The risk factors of indicators and hyperlipidemia indicators are examined [11–13]. Studies have demonstrated that individuals with ONFH commonly exhibit inflammatory reactions. Consequently, there is evidence to suggest that the administration of pharmacological interventions can effectively ameliorate the inflammatory response. The examination of inflammatory markers as a risk factor for group statistical analysis has also been explored in previous studies [14, 15]. In recent research, it has been observed that the administration of glucocorticoids might contribute to the development of fatty liver, hence impacting liver metabolism. Additionally, excessive alcohol consumption, known as a risk factor for ONFH, can also induce liver steatosis and impair liver function. Indices of hepatic metabolism for group analysis [16–18]. Several studies have indicated that the BMI has the potential to influence ONFH. Additionally, it has been observed that obesity might impact bone metabolism, hence potentially exacerbating the occurrence of ONFH [5, 19].

### ONFH Patients with age and gender distribution

The statistical analysis conducted on patients with ONFH revealed that the age distribution of the patients was as follows: individuals younger than 45 years old, those aged 45–60 years old (odds ratio [OR]: 2.534, 95% confidence interval [CI]: 2.082–3.090, *P* < 0.001), and individuals aged 60 years or more (OR: 5.735, 95% CI: 4.467–7.402, *P* < 0.001). Patients who are at a greater risk of developing the condition exhibit a larger likelihood of onset in the age groups of 45–60 years and over 60 years, as compared to those who are 45 years old. The majority of individuals in this cohort are classified as inpatient patients, primarily falling under ARCO stages 3 or 4. It is important to note that these patients are experiencing illness. The majority of the observed period occurred around two to three years before to the present investigation, resulting in a notable prevalence of individuals aged 45 to 60 years [1].

In relation to gender, the number of male patients exceeds that of female patients. The study found a statistically significant association between male gender and the outcome variable (OR: 1.310, 95%CI: 1.106–1.554, *P* = 0.002). The incidence rate is elevated, with a patient ratio of approximately 1.25:1, as observed in China throughout the year 2020. The ratio of 2.4:1 exhibited by the team led by Wengheng Chen, as well as the ratio of 2.4:1 seen in the study conducted by Kang et al., are of interest. In a study conducted in 2009, researchers [5, 20] postulated the presence of a potential divergence in their findings. It should be noted that this speculation is based solely on the data collected from patients at the first hospital in Gansu Province.

### ONFH patient BMI distribution

Obesity is recognized as a significant contributing factor in osteonecrosis ONFH. Extensive research has indicated that obesity might potentially impact bone metabolism and increase the risk of developing osteoporosis, hence leading to fractures [21, 22]. This study revealed that those with a BMI more than 24 had a higher chance of developing the condition compared to those with a BMI below 24 (odds ratio [OR]: 1.540, 95% confidence interval [CI]: 1.302–1.823, *P* < 0.001). Based on the obtained findings, it is plausible to suggest that an elevated BMI value may serve as a potential risk factor for ONFH. Regarding the correlation between obesity and ONFH, it is important to include additional factors such as hyperlipidemia. However, further investigation is required to confirm this relationship [23–25].

### ONFH patient laboratory index distribution

Previous research has indicated that thrombosis is a significant risk factor for ONFH. Additionally, several data suggest that elevated levels of DD and fibrinogen (FIB) may contribute to the formation of thrombosis, hence exerting a substantial influence on the development of ONFH [26–28]. In recent research, it has been demonstrated that elevated PLT can potentially serve as a preventive measure against hormonal disruptions and the subsequent occurrence of femoral head necrosis [29, 30]. In recent times, there has been a surge in the utilization of platelet-rich plasma as a therapeutic approach for the management of femoral head necrosis. According to previous studies [31, 32], hypertension has been found to potentially enhance the development of blood vessels and promote the differentiation of osteocytes. There is a paucity of research examining the association between IBIL and JBCR in the context of ONFH. Further experimentation in the future is warranted to refine the intervention strategies of IBIL and JCBR for the treatment of osteonecrosis ONFH. Several studies have demonstrated a positive correlation between excessive alcohol consumption and an elevation in γ-GT activity associated with ONFH. Hence, γ-GT has a specific sensitivity in the identification of alcohol-induced ONFH. Additionally, this study provides evidence supporting a positive correlation between γ-GT levels and the presence of ONFH [33, 34]. Previous research has posited a negative association between HDL-C and APOA with ONFH. This experiment additionally demonstrates a negative correlation between HDL-C and APOA1 levels and patients diagnosed with ONFH. The provided text consists of a numerical range.

The results of a univariate analysis indicate that several indicators possess statistical significance. However, to gain a more comprehensive understanding of the age disparity between the two patient groups and the variations in BMI, it is recommended to conduct further investigations employing the receiver ROC curve and doing a specific analysis on the Asian subgroup. The AUC for the inflammatory cell indicators in the JBCR is 0.605. For the coagulation index, the AUC values for the DD and FIB are 0.738 and 0.668, respectively. In the liver metabolic index, the AUC value for IBIL is 0.628. Lastly, the AUC value for the hyperlipidemia indicator HDL-C is also included. The correlation coefficient of 0.602 between the variable in question with the commencement of the condition indicates a stronger association, suggesting that it may have some potential relevance in guiding the diagnosis of ONFH. However, accurately assessing the diagnostic effectiveness of this variable, apart from using DD, poses challenges. Upon doing a more comprehensive examination of the Asian population, it was discovered that the inflammatory cell markers within each respective Asian subgroup, specifically JBCR, PLT, IBIL, and HDL-C, had significant associations with the susceptibility to the onset of Essence.

### Insufficient and outlook of this experiment

This study, while analyzing the risk factors of ONFH over 6 years in a single center and being rooted in real-world clinical observations, has its set of constraints: Given the retrospective nature of this research, the majority of our patients were in the advanced stages as per ARCO classification, with fewer in the early stages. Although we had a reasonably large sample size, age disparities were evident. Due to various challenges in real-world studies, there was an inconsistent collection of cases across patients. Also, given the vast individual differences, we could not delve deeply into basic diseases for each patient. Of the 2202 patients, 450 used corticosteroids, 380 consumed alcohols regularly, and 520 had other ON risk exposures. These factors could influence ONFH, and the markers studied. However, we did not stratify our analysis based on these factors, acknowledging it as a limitation. Animal studies are vital for understanding specific laboratory risk indicators for early ONFH diagnosis. We also aim to expand our sample in subsequent research for more accurate data.

## Conclusion

Femoral head necrosis is associated with a range of variables including coagulation malfunction, lipid metabolic abnormalities, and inflammation.

## Abbreviations

WBC: White blood cell
MBCR: Carbon dioxide combining power
LBCR: Lymphocyte ratio
DBCR: Monocyte ratio
SBCR: Eosinophil ratio
JBCR: Basophil ratio
PLT: Platelet
HCY: Homocysteine
FDP: Fibrinogen degradation products
DD: D-dimer
APTT: Activated partial thromboplastin time
PT: Prothrombin time
TBIL: Total bilirubin
DBIL: Direct bilirubin
IBIL: Indirect bilirubin
ALT: Alanine aminotransferase
AST: Aspartate aminotransferase
AST/ALT: Aspartate/alanine
Γ-GT: *γ*-glutamyl transpeptidase
ALP: Alkaline phosphatase
AMY: Amylase
CHO/HDL: Very low-density lipoprotein
APOA1: Apolipoprotein1
APOB: Apolipoprotein B
TCHO: Total cholesterol
HDL-C: High-density lipoprotein cholesterol
LDL-C: Low-density lipoprotein cholesterol
VLDL: Very low-density lipoprotein cholesterol
TG: Triglyceride
